# Oocyte degeneration in *Pygocentrus nattereri* induced by *Myxobolus* sp. (Cnidaria: Myxozoa) in the Brazilian Amazon

**DOI:** 10.1590/S1984-29612025057

**Published:** 2025-10-24

**Authors:** Antônio Carlos de Oliveira Souza, Nayana Moraes de Sena, Camila Maria Barbosa Pereira, Jhonata Eduard, Michele Velasco, José Ledamir Sindeaux-Neto

**Affiliations:** 1 Universidade Federal Rural da Amazônia – UFRA, Laboratório de Integração Morfo-Molecular e Tecnologias – LIMT, Instituto de Saúde e Produção Animal – ISPA, Belém, PA, Brasil; 2 Universidade Federal Rural da Amazônia – UFRA, Programa de Pós-graduação em Reprodução Animal na Amazônia – REPROAMAZON, Belém, PA, Brasil; 3 Universidade Federal Rural da Amazônia – UFRA, Programa de Pós-graduação em Saúde e Produção Animal na Amazônia – PPGSPAA, Belém, PA, Brasil; 4 Universidade Federal do Pará – UFPA, Programa de Pós-graduação em Biodiversidade e Biotecnologia, Belém, PA, Brasil; 5 Universidade Federal do Pará – UFPA, Programa de Pós-graduação em Biologia de Agentes Infeciosos e Parasitários – PPGBAIP, Instituto de Ciências Biológicas – ICB, Belem, PA, Brasil

**Keywords:** Red piranha, *Myxobolus* sp., parasitic infection, reproductive health, Amazon, Piranha vermelha, *Myxobolus* sp., infecção parasitária, saúde reprodutiva, Amazônia

## Abstract

The red piranha (*Pygocentrus nattereri*) is a carnivorous fish of significant ecological and economic value, widely distributed across tropical and neotropical regions of South America, particularly within the Amazon basin. This study investigated the presence of *Myxobolus* sp. infection in the ovaries of red piranhas collected in the municipality of Pracuúba, in the state of Amapá, Brazil. During necropsy, whitish cysts suggestive of parasitic infection were observed. Microscopic examination revealed spores with morphological characteristics consistent with *Myxobolus* sp., predominantly located in the ovarian parenchyma. These infections caused tissue lesions and disrupted the normal ovarian architecture, although no inflammatory infiltrates were detected. While *Myxobolus* sp. infections in reproductive organs are rare, they can significantly impact fish health and reproduction. This study is the first to report of *Myxobolus* sp. infection in the ovaries of *P*. *nattereri*, underscoring the importance of monitoring parasitic infections in this species, which plays a vital role in the food security of riverside communities in the Amazon.

## Resumo

A piranha vermelha (*Pygocentrus nattereri*) é um peixe carnívoro de significativo valor ecológico e econômico, amplamente distribuído pelas regiões tropicais e neotropicais da América do Sul, particularmente na bacia amazônica. Este estudo investigou a presença de infecção por *Myxobolus* sp. nos ovários de piranhas vermelhas coletadas no município de Pracuúba, no estado do Amapá, Brasil. Durante a necropsia, foram observados cistos esbranquiçados sugestivos de infecção parasitária. O exame microscópico revelou esporos com características morfológicas consistentes com *Myxobolus* sp., predominantemente localizados no parênquima ovariano. Essas infecções causaram lesões nos tecidos e interromperam a arquitetura ovariana normal, embora nenhum infiltrado inflamatório tenha sido detectado. Embora as infecções por *Myxobolus* sp. nos órgãos reprodutivos sejam raras, elas podem impactar significativamente a saúde e a reprodução dos peixes. Este estudo é o primeiro a relatar *Myxobolus* sp. causando infecção nos ovários de *P. nattereri*, ressaltando a importância do monitoramento de infecções parasitárias nesta espécie, que desempenha um papel vital na segurança alimentar das comunidades ribeirinhas da Amazônia.

## Introduction

*Pygocentrus nattereri* Kner, 1860 (red piranha), is a serrasalmid fish with obligate carnivory, broadly distributed across tropical and neotropical South America. In Brazil, this species predominates in the Amazon Basin but also inhabits the Tocantins-Araguaia, São Francisco, and Paraná river systems (Manna et al., 2013).

As both predator and scavenger, *P. nattereri* occupies a pivotal ecological niche in freshwater food webs and fulfills an important socioeconomic role (Manna et al., 2013). Riversider populations in northern Brazil rely on it as a subsistence fishery, and it is marketed locally in Amazonian municipalities (Vicentin et al., 2013).

Despite its resilience, *P. nattereri* is vulnerable to a variety of parasitic infections, notably by myxosporeans of the genus *Myxobolus*-one of the most speciose groups of fish parasites (Maor-Landaw et al., 2023). Myxosporean infections can afflict multiple organs (e.g., muscle, kidney, liver, gonads, nervous system), leading to lesions that impair growth, reproduction, and survival in both freshwater and marine hosts (Maor-Landaw et al., 2023). The genus *Myxobolus* exhibits global distribution and high host diversity, with new species described regularly from varied aquatic habitats (Xu et al., 2025).

Although myxosporeans parasitism has been documented in many fish taxa, studies focusing on piranhas–particularly *P. nattereri*-are scarce. Silva et al. (2018) were among the first to document a myxosporean in this species, reporting *Ellipsomyxa arariensis* in the swim bladder. Even fewer accounts describe myxosporean infections in the gonads of *P. nattereri*, Stilwell et al. (2020) remain one of the only sources to note such occurrences.

Because *P. nattereri* represents a key protein source for riverside communities through the Amazon, undrstanding the prevalence and pathology of its parasitic infections is crucial. Such information will inform assessments of fish health and the potential implications for regional food security. Here, we report histopathological evidence of *Myxobolus* sp. infection in the ovaries of red piranhas collected in Pracuúba, Amapá, Brazil.

## Material and Methods

### Necropsy and parasitological examination

Specimens were collected from Lake Sacaizal (1°42'8.79”N, 50°43'17.56”W) in the municipality of Pracuúba, Amapá, Brazil. Eighteen *Pygocentrus nattereri* individuals were obtained postmortem from artisanal fishermen, under SISBIO/ICMBio License No. 88196-1. Upon collection, each specimen was placed in an insulated boxes with ice and transported to the Laboratory of Integration and Morphomolecular Technologies (LIMT) at the Federal Rural University of the Amazon (UFRA), Belém, Pará. Standard necropsy procedures were then performed, including a thorough external examination and systematic dissection of all major organs, with particular attention to the gonads for evidence of parasitism.

The study protocol was approved by the Ethics Committee on the Use of Animals (CEUA No. 7218270723/ID000609). Fish exterior surfaces, tissues, and internal organs were systematically examined under stereoscopic and compound light microscopy to detect parasites stages and performed detailed morphological analyses. Genus-level identification of myxozoans followed the diagnostic criteria of Lom & Dyková (2006).

### Histopathological analysis

Upon detection of parasitic cysts, small fragments of ovarian tissue were excised and fixed in Davidson’s solution (95% ethanol, 37-40% formaldehyde, acetic acid, and distilled water) for 24 hours. Fixed samples were then processed for paraffin embedding: they were dehydrated through a graded ethanol series, cleared in xylene, and embedded in paraffin wax. From each paraffin block, 5 µm sections were cut and mounted on mounted on glass slides, which were incubated at 37^o^C for 24 hours to ensure complete removal of residual paraffin. Before staining, slides were deparaffinized in xylene and rehydrated through descending ethanol concentrations. Sections were then stained by the Ziehl-Neelsen method following Luna (1968) and examined under a compound light microscope for detailed histopathological evaluation.

## Results

**Figure 1:**
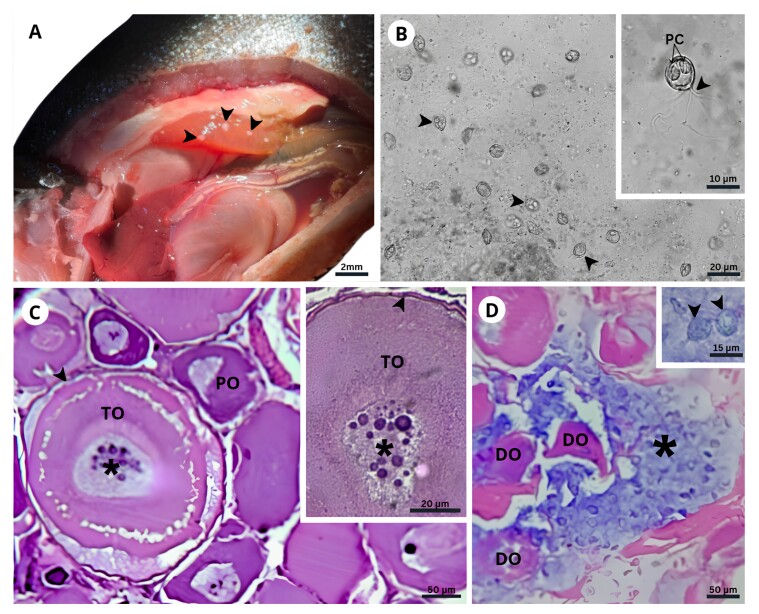
A- Macroscopic image of a dissected *Pygocentrus nattereri* specimen showing whitish cysts (arrowheads) in the gonadal tissue. B- Light photomicrograph of *Myxobolus* sp. myxospores (arrowheads) located within ovarian cysts. C- Histological section of *P. nattereri* ovary stained with Ziehl-Neelsen, showing rounded cysts containing spores suggestive of *Myxobolus* sp. (*) within tertiary oocytes (TO). Several apparently non-parasitized oocytes, including primary oocytes (PO), are also visible. Inset: Spores (*) located in the central region of a tertiary oocyte (TO) with a preserved follicular capsule (arrow). D- Histological section of *P. nattereri* ovary stained with Ziehl-Neelsen, showing *Myxobolus* sp. myxospores (*) dispersed throughout the ovarian stroma, with oocytes undergoing advanced degeneration (DO). Inset: Myxospores with morphology consistent with those observed in fresh preparations, characteristic of the genus *Myxobolus* (arrowhead), located within oval parasite cysts, indicating an ongoing inflammatory process.

Necropsy of 18 red piranha specimens revealed spherical, whitish cysts in the ovaries of one individual (5.5%; 1/18), indicative of parasitic infection (Figure 1A). Macroscopic lesions prompted microscopic examination of the gonadal tissue, which revealed spores morphologically consistent with *Myxobolus* sp., predominantly localized within the ovarian parenchyma. Several spores displayed atypical posterior extensions (Figure 1B). Characteristically, *Myxobolus* sp. spores are round, ovoid, or pyriform and contain two polar capsules and two shell valves.

Microscopic examination revealed rounded cysts within mature oocytes (Figure 1C) and dispersed throughout the ovarian stroma (Figure 1D), indicative of oocyte rupture. Intra-oocytic lesions were characterized by disruption of normal oocyte architecture and central accumulation of spores. Severely degenerated oocytes exhibited multifocal breakdown, often association with free spores in the surrounding stroma. Notably, despite extensive tissue damage, there was no observable inflammatory cell infiltration.

## Discussion

This study reports the presence of *Myxobolus* myxospores in the ovaries of *P. nattereri*-an uncommon infection site for this genus, which typically exhibits tropism for the gills of its hosts, as noted in several taxonomic reviews (Eiras et al., 2005, 2014, 2021). Ovarian infections by *Myxobolus* sp. are rarely documented. Notable exceptions include Gbankoto et al. (2015), who reported *Myxobolus* sp. infecting the ovarian follicles of *Clarias gariepinus*, and Xu et al. (2025), who provided morphological and molecular characterization of a novel species, *M. aculeatus*, found in the ovaries of *Macrognathus aculeatus*. In Brazil, *Myxobolus batalhensis* remains the only species described to infect the ovaries and liver of *Salminus hilarii* (Vieira et al., 2017).

The 5.5% prevalence of *Myxobolus* sp. observed in this study is relatively low compared to reports of infections in the same anatomical site in other fish species. For instance, *M. algonquinensis* was recorded in the ovaries of *Notemigonus crysoleucas* at a prevalence of 15% (Xiao & Desser, 1997), *M. dahomeyensis* in *Tilapia zillii* at 31.6%, and *M. batalhensis* in *Salminus hilarii* at 62.5% (Grankoto et al., 2001).

*Pygocentrus nattereri* is an ornamental species. Interestingly, infections caused by *Myxobolus* have been reported in recent decades, with the description of new species such as *Myxobolus lentisturalis* and *M. branchioepidermis* in *Carassius auratus* from Iran (Rahmati-Holasoo et al., 2024, 2025). In South America, records include *M. iquitoensis* in *Otocinclus cocama* from Peru, and *M*. *adrianoi* in *Corydoras schwartzi* as well as *M. matogrossoensis* in *Hyphessobrycon eques*, both described in the Brazilian Pantanal (Mathews et al., 2020a, b, 2022).

Histopathological analysis revealed that *Myxobolus* sp. myxospores were predominantly located within the ovarian stroma, consistent with findings reported for *M. aculeatus* infection by Xu et al. (2025). However, the inflammatory response observed in that study was less pronounced than in the present investigation, indicating that the severity of inflammation and the intensity of infection may vary across fish species. A notable distinction in the current study was the widespread distribution of myxospores throughout the ovarian tissue of *P. nattereri*. Although the histological lesions shared certain similarities, the infection dynamics and parasite behavior appear to be influenced by host-specific factors-particularly those related to the immune response (Alho et al., 2015).

In general, gonadal infections caused by myxozoans elicit histopathological alterations that can significantly impair host reproductive function, potentially leading to gonadal regression, reduced fecundity, and, in severe cases, infertility. Gbankoto et al. (2015) reported that *Myxobolus* sp. infection in the ovaries of the catfish *Clarias gariepinus* resulted in dissolution, deformation, and functional disruption of oocyte internal structures, ultimately leading to reproductive failure. Similar pathological changes were observed in the present study; however, it was not possible to determine whether infertility occurred in *P. nattereri*, as numerous oocytes retained normal morphology and exhibited characteristics indicative typical developmental stages.

Zhou et al. (2025) demonstrated that *Myxobolus honghuensis* infecting the oocytes of female goldfish (*Carassius gibelio*) was transmitted to the embryos, providing evidence of vertical transmission. This mode of transmission is unusual, as the majority of myxozoans exhibit horizontal transmission, which requires an intermediate host, such as an annelid or bryozoan, to complete their life cycle (Okamura et al., 2015). Considering the marked pathogenicity of *Myxobolus* sp. infections observed in the oocytes of *Pygocentrus nattereri*, further investigations are warranted to evaluate the potential occurrence of vertical transmission in this host.

It is important to highlight that infections affecting the reproductive systems of fish, with the potential to threaten the sustainability of natural stocks in the Brazilian Amazon, pose serious ecological and socioeconomic concerns (Alho et al., 2015). The red piranha is among the most commonly consumed fish species in Northern Brazil, playing a crucial role in the diet and subsistence of riverine populations, while also contributing directly to the local economy, particularly in regional markets (Sipos et al., 2018). Therefore, any health-related impacts-such as parasitic infections-may compromise food security and undermine the long-term sustainability of fisheries that are vital to these communities (Morais et al., 2019).

## Conclusion

This study represents the first documented case of *Myxobolus* sp. infection in the ovaries of piranha fish, a novel finding given the absence of prior records for this host species in the scientific literature. Histopathological analysis revealed cysts containing *Myxobolus* sp. spores localized within the ovarian stroma, accompanied by tissue degeneration. Although infections *Myxobolus* of reproductive organs by *Myxobolus* sp. are relatively rare, their potential impact on reproductive success may be considerable. These findings underscore the importance of monitoring the health status of *P. nattereri* and the parasites that affect this species, in order to inform strategies aimed at minimizing potential economic losses and ecological disruptions in the Amazon region.

## Data Availability

The data that support the findings of this study are available from the corresponding author upon request.
